# Transcription factors involved in the regulation of natural killer cell development and function: an update

**DOI:** 10.3389/fimmu.2012.00319

**Published:** 2012-10-15

**Authors:** Martha Luevano, Alejandro Madrigal, Aurore Saudemont

**Affiliations:** ^1^Anthony Nolan Research InstituteLondon, UK; ^2^University College LondonLondon, UK

**Keywords:** natural killer cells, development, maturation, transcription factors, regulation of gene expression

## Abstract

Natural killer (NK) cells belong to the innate immune system and are key effectors in the immune response against cancer and infection. Recent studies have contributed to the knowledge of events controlling NK cell fate. The use of knockout mice has enabled the discovery of key transcription factors (TFs) essential for NK cell development and function. Yet, unwrapping the downstream targets of these TFs and their influence on NK cells remains a challenge. In this review, we discuss the latest TFs described to be involved in the regulation of NK cell development and maturation.

## INTRODUCTION

Natural killer (NK) cells are innate immune lymphocytes that play an important role in the immune response against tumors and infection by either mediating direct cell lysis or by producing a plethora of cytokines (Trinchieri, 1989). The bone marrow (BM) is considered to be the main site of NK cell development, however, it has been reported that NK cell development and maturation can take place in other sites such as the thymus (Vosshenrich et al., 2006) and lymph nodes (LNs; Freud et al., 2005, 2006). NK cell development occurs in different stages, CD34^+^ hematopoietic stem cells differentiate into common lymphoid progenitors that can give rise to NK cell precursors that then develop into immature NK cells. Finally, the maturation of immature NK cells into fully functional NK cells involves the acquisition of activating and inhibitory receptors that regulate NK cell effector functions (Lanier, 2005). NK cell development has been the subject of many studies, major contributions have been made during the last decade and several pathways of NK cell development have emerged, however, our understanding of how these developmental stages are regulated on a molecular level is still relatively limited in mice and even more so in humans (Hesslein and Lanier, 2011; Martin-Fontecha et al., 2011).

**FIGURE 1:**
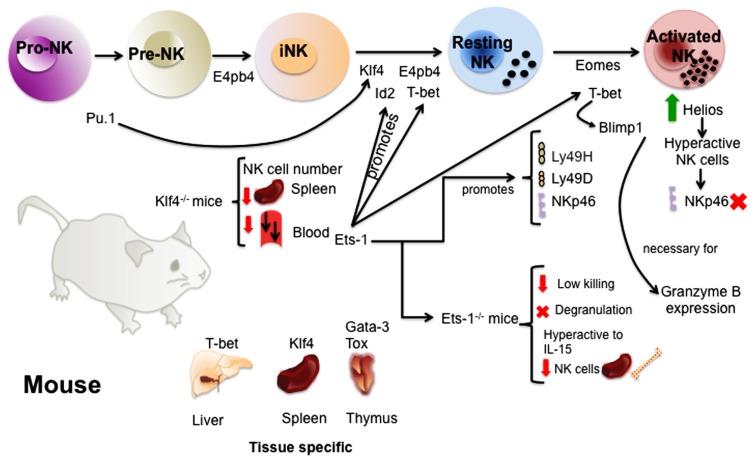
**Transcription factors involved in the regulation of murine NK cell development and maturation.** The study of TFs in mice has given valuable information about the molecular regulation of NK cell development. TFs like E4bp4 and Pu.1 act early promoting NK cell lineage commitment. Then, several TFs such as E4bp4, Id2, Klf4, and T-bet ensure the transition from immature to mature NK cells. A complex network of TFs act downstream or upstream of these TFs and others regulating the acquisition of receptors and NK cell function. Finally, some of the TFs like Gata-3, Klf4, T-bet, and Tox have been shown to be important for the regulation of tissue-specific NK cells.

Transcription factors (TFs) are key molecules in the regulation of gene transcription and have a great influence on immune cell fate and differentiation. Knockout mice have allowed the identification of several TFs involved in the commitment to the NK cell lineage, in the regulation of NK cell maturation or of tissue-specific NK cells. This review summarizes the latest information on the role of TFs in the regulation of NK cell development, maturation, and function in both mice (**Figure 1**) and humans.

## TRANSCRIPTION FACTORS INVOLVED IN THE REGULATION OF NK CELL DEVELOPMENT

### E4BP4

E4-binding protein 4 (E4BP4), also called nuclear factor interleukin-3 (NFIL3), is a basic leucine zipper TF shown to regulate a broad range of mechanisms such as the mammalian circadian clock (Mitsui et al., 2001) and IL-3-dependent survival of pro-B cells (Ikushima et al., 1997) to name some. Using mice deficient for E4bp4, Gascoyne et al. (2009) and Kamizono et al. (2009) demonstrated that E4bp4 is required for NK cell development and maturation. They showed that E4bp4-deficient mice specifically lacked mature NK cells, and no other hematopoietic cell lineages, and also had a low number of immature NK cells, suggesting that E4bp4 is essential for the progression from NK cell precursors to immature NK cells and then from immature NK cells to mature NK cells. To note that both studies also reported that E4bp4-deficient NK cells exhibited impaired cell cytotoxicity and low IFN-γ production. Moreover, Gascoyne et al. (2009) also demonstrated that E4bp4 acted downstream of the interleukin 15 receptor (IL-15R) while the TF Id2 was downstream of E4bp4. These studies identified for the first time a gene that specifically determined the NK cell lineage.

### ID TRANSCRIPTION FACTORS

Helix-loop-helix TFs of the Id family are important regulators of cellular differentiation. Id proteins are transcriptional repressors that antagonize E proteins activity by binding to these proteins, which in turn are unable to associate with E box sequences in DNA (Sun et al., 1991). E proteins are TFs that can either form homodimers and act as transcription activators or form heterodimers with some basic helix-loop-helix proteins and then act as transcription activators or repressors (Kee, 2009). E proteins are able to bind the Id family proteins Id1–4. In addition, others targets than E proteins have been suggested for Id2 such as the retinoblastoma protein (Iavarone et al., 1994) but E proteins are their main binding partners.

It is known since the late 1990s that Id proteins are essential for NK cell development and maturation. Mice deficient for Id2 have been shown to lack LNs, Peyer’s patches as well as to have reduced number of mature NK cells in the spleen (Yokota et al., 1999). Furthermore, it was reported that the overexpression of Id3 in CD34 progenitors was able to inhibit T cell development and to favor the generation of NK cells (Heemskerk et al., 1997). The role of Id2 in the regulation of NK cell development was confirmed by another study from Boos et al. (2007), although the role of Id2 seems to be essential at a later stage in differentiation. They reported that Id2^– / –^ mice have low number of mature NK cells in the periphery while having normal number of immature NK cells and NK cell precursors in the BM highlighting the importance of Id2 for the maturation of immature NK cells. The phenotype observed in Id2^– / –^ animals was linked to the capacity of Id2 to inhibit E2A proteins.

## TRANSCRIPTION FACTORS INVOLVED IN THE REGULATION OF NK CELL MATURATION AND FUNCTION

### BLIMP1

BLIMP1, also known as PRMD1 or PRDI-BF1, is a transcriptional repressor crucial for the terminal differentiation of plasma cells and CD8^+^ effector T cells. Mouse NK cells all express one isoform of Blimp1 (Kallies et al., 2011). Blimp1 expression is induced by IL-15 during differentiation but can be upregulated by cytokines during maturation, the most mature NK cells expressing the highest level of BLIMP1 in both humans and mice. BLIMP1 is necessary for NK cell peripheral maturation and homeostasis but is not required to obtain normal number of NK cells in mice. While IRF4 and Bcl6 regulate Blimp1 expression in B and T cells, it has been shown that T-bet acts upstream of Blimp1 in mouse NK cells. Finally, cytokine production and cytotoxicity is not altered in Blimp1-deficient NK cells, however, Blimp1 was essential for high granzyme B expression in NK cells.

Human NK cells, in particular CD56^dim^ NK cells, express three distinct isoforms of BLIMP1 (α, β, and a splice variant) upon activation (Kallies et al., 2011), BLIMP1β being the predominant isoform present in human NK cells. Whereas BLIMP1 did not seem to be essential for the regulation of NK cell function in mice, BLIMP1 negatively regulate the production of IFN-γ, TNF-α, and TNF-β in human NK cells highlighting a differential role of BLIMP1 in mouse and human NK cells. As for mouse NK cells, BLIMP1 does not affect human NK cell cytotoxicity.

### EOMES

In vertebrates, as for T-bet, Eomesodermin (Eomes) belongs to the T-box family of TFs and plays an important role in the initiation of mesodermal differentiation and in determining cell fate (Ryan et al., 1996). In addition, Eomes has been reported to be a key factor controlling CD8^+^ T cell lytic function (Pearce et al., 2003) by regulating granzyme B and perforin expression (Sullivan et al., 2003). In mice, Eomes is essential for NK cell maturation and it has been shown that Eomes is necessary to maintain the mature attributes of NK cells (Gordon et al., 2012).

Gill et al. (2012) in an effort to elucidate the mechanisms of NK cell exhaustion after adoptive transfer, discovered that T-bet and Eomes are rapidly downregulated in NK cells upon transfer in tumor bearing mice. There was an inverse correlation between NK cell proliferation and IFN-γ production and the expression of these TFs. Overexpression of Eomes, not T-bet, resulted in tumor burden reduction (Gill et al., 2012) and Eomes expression was not affected by T-bet expression (Townsend et al., 2004).

Finally, the work by Gordon et al. (2012) suggests that T-bet and Eomes regulate two sequential checkpoints of NK cell maturation. Additional research will help to clarify the role of these TFs, since other authors suggest that adoptive transfer experiments did not provide sufficient evidence that T-bet and Eomes act sequentially on NK cell maturation (Jiang et al., 2012).

### ETS-1

ETS-1 belongs to the Ets family of winged helix-turn-helix TFs. While Elf-1, another TFs of the Ets family, is not required for NK cell development (Choi et al., 2011), it has been known for more than a decade than Ets-1-deficient mice have a reduced number of NK cells notably in the spleen and BM (Barton et al., 1998), however, the exact function of Ets-1 in NK cells and when Ets-1 is important for NK cell development has only been discovered recently.

Natural killer cell development and NK cell function are severely impaired in Ets-1-deficient mice. It has been shown that Ets-1 is a key TFs very early during NK cell development in particular to induce the expression of other TFs also involved in the regulation of NK cell development such as T-bet and Id2 (Ramirez et al., 2012). Moreover, Ets-1 is also essential for the expression of key receptors that regulate NK cell activity such as NKp46, Ly49H, and Ly49D in mice. Barton et al. (1998) reported that Ets-1-deficient NK cells were poorly cytolytic and were not able to kill YAC1 cells. Ramirez et al. (2012) report that NK cells deficient for Ets-1 are not able to degranulate after stimulation and express lower levels of NK cell receptors, which could explain Ets-1-deficient NK cell low cytotoxic activity. However, Ets-1-deficient NK cells were hyper-responsive to cytokines such as IL-15 and exhibited characteristics of chronic activation such as increased expression of inhibitory receptors.

### GATA-3

GATA-3 is a TF that plays an important role in T cell development and T cell differentiation (Ting et al., 1996; Zheng and Flavell, 1997). Even though the number of NK cells was reported to be normal in the spleen of Gata-3-deficient mice (Samson et al., 2003), NK cells in these mice were actually immature suggesting a role for Gata-3 in NK cell maturation but not in NK cell specification. NK cell cytotoxicity was not affected in the absence of Gata-3, however, Gata-3-deficient NK cells produced less IFN-γ than wild-type (WT) mice. This study also showed that Gata-3 was important for NK cell response to *Listeria monocytogenes* infection. Moreover, Gata-3 was demonstrated to be involved in the regulation of NK cell homeostasis as the number of NK cells was drastically reduced in the liver of Gata-3^– / –^ mice. Finally, Vosshenrich et al. (2006) reported that although Gata-3 was dispensable for the development of NK cells in the BM it was indispensable for the generation of CD127^+^ thymic NK cells.

### HELIOS

Helios is a member of the Ikaros family of TFs. The role of this TFs has been studied mainly in regulatory T cells (Getnet et al., 2010) and lymphoid malignancies (Rebollo and Schmitt, 2003). It has been shown that Helios can be induced during T cell activation and proliferation (Akimova et al., 2011).

Using the mice model *Noe*, Narni-Mancinelli et al. (2012) found that the absence of NKp46 expression made NK cells hyperactive. The study of the transcriptome of WT mice revealed the increased expression of the TF Helios in the mature CD11b^+^ NK cell subset expressing NKp46. In *Noe* mice, Helios transcripts were twice as abundant in the CD11b^+^ NK cells as compared to the same subset in WT mice. Silencing of Helios in NK cells isolated from *Noe* mice restored their reactivity to the level observed for WT NK cells. The authors suggest that Helios downregulation is involved in the regulation of NK cell reactivity via NKp46 (Narni-Mancinelli et al., 2012).

### KLF4

Krüppel-like factor 4 (KLF4) is a TF key in the regulation of stem cell pluripotency. Klf4 is a downstream target of Pu.1 and is an important TF that determines the progenitor cell fate of different immune cells such as NK cells. Using inducible and lineage-specific Cre transgenic mice, it was reported that the loss of Klf4 resulted in low numbers of NK cells in the blood and in the spleen but normal numbers in other organs such as the BM, liver, and LNs (Park et al., 2012). These mice also exhibited increased apoptosis of NK cells in the spleen but the remaining NK cells were fully functional. This defect was not intrinsic as adoptive transfer of Klf4-deficient NK cells in WT mice shows recovery of the phenotype. As the number of conventional dendritic cells was lower in the spleen of Klf4-deficient animals it was suggested that Klf4 is essential for dendritic cell maintenance in the spleen promoting NK cell survival in that organ.

### T-bet

T-bet (Tbx21) belongs to the T-box family of TFs, involved in the early cell fate decision, cell differentiation, and organogenesis (Wilson and Conlon, 2002). Knockout mice have given valuable insight into the role of this TF in the regulation of immune cells. T-bet was first described as an initiator of T helper (Th)1 lineage development, redirecting Th2 and Tc2 primary T cells into the Th1 lineage, controlling the generation of CD8^+^ cytotoxic effector cells, and the expression of IFN-γ into those cells (Szabo et al., 2000; Sullivan et al., 2003).

Moreover, T-bet-deficient mice exhibited a reduced number of NK cells in the spleen, liver, and peripheral blood (Townsend et al., 2004). T-bet^– / –^ NK cells showed a high expression of cKit and α_v_ integrin, markers of immature NK cells. The detection of high levels of CD69 suggested an activated state of these NK cells. In addition, these hyperactivated cells underwent augmented spontaneous apoptosis. Finally, T-bet^– / –^ NK cells showed impaired cytotoxicity and IFN-γ production in response to murine cytomegalovirus highlighting a critical role for T-bet in the control of NK cell maturation (Townsend et al., 2004).

The proximal promoters of T-BET contain two Ets binding sites that are highly conserved. ETS TFs such as MEF, PU.1, and ETS1 can probably regulate the expression of T-BET during the last NK cell development stages (Townsend et al., 2004). It has also been suggested that T-BET expression can be regulated by GATA-3 (Samson et al., 2003) and indirectly by TOX (Yun et al., 2011).

In addition, it has been suggested that T-bet expression in NK cells is important for the control of metastatic disease (Werneck et al., 2008) and the crosstalk between the innate and adaptive immunity. In this study, T-bet^– / –^ NK cells had reduced longevity *in vivo* when compared to WT NK cells, their apoptotic phenotype and impaired effector function, low IFN-γ secretion and low killing *in vitro*, correlated with the susceptibility of T-bet^– / –^ mice to develop melanoma metastasis.

Furthermore, T-bet regulates the expression of sphingosine-1 phosphate receptor 5 (S1P5) that plays an important role in NK cell recirculation (Walzer et al., 2007). Duane, a mouse strain with a point mutation in T-bet, has a decreased number of circulating and spleen NK cells. Duane mice have an altered NK cell trafficking, with NK cell accumulation in LNs and BM (Jenne et al., 2009), identifying T-bet driven S1P_5_ expression to be essential for NK cell egress from LNs and BM.

Finally, Gordon et al. (2012) noticed the absence of the immature population expressing the death receptor TRAIL (TRAIL^+^ DX^-^), found normally in the liver of WT mice. When T-bet was temporarily deleted *ex vivo* in hepatic NK cells and transferred to immunodeficient mice, only TRAIL^-^ NK cells were found. This suggests that T-bet plays a role in the maintenance of the TRAIL^+^ subset (Gordon et al., 2012). In mice, NK cells undergo four development stages according to the expression of CD11b and CD27 (CD11b^low^CD27^low^ → CD11b^low^CD27^high^ → CD11b^high^CD27^high^ → CD11b^high^CD27^low^; Chiossone et al., 2009). Gordon et al. (2012) proposed that T-bet is necessary for CD27 repression among mature NK cells. The authors found that this repression was cell intrinsic, contrary to Soderquest et al. (2011), that suggest that the transition from CD11b^high^CD27^high^ to CD11b^high^CD27^low^ could also be partially extrinsic as the expression of T-bet and IL-15Rα on monocytes is determinant to allow NK cells to proceed to the final differentiation stage.

**FIGURE 2:**
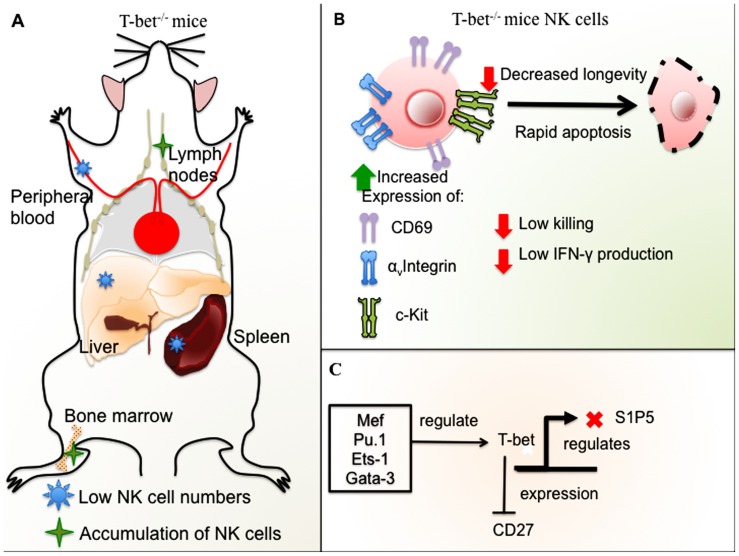
**Role of T-bet in NK cell development and function in mice. (A)** T-bet-deficient mice have low NK cell numbers in the spleen, liver, and peripheral blood with accumulation of NK cells in LNs and BM. **(B)** NK cells from T-bet-deficient mice have an immature phenotype, expression of α_v_ integrin and ckit, and high levels of CD69. T-bet^– / –^ NK cells undergo rapid apoptosis and have decreased longevity in addition to impaired cytotoxicity and low IFN-γ production. **(C)** Different TFs regulate T-bet, among these, Ets-1, Gata-3, Mef, and Pu.1. T-bet represses CD27 expression in mature NK cells and negatively regulates S1P5.

Altogether, the recent research suggests that T-bet is a key factor that controls NK cell maturation and cytokine, perforin, and granzyme B production in NK cells. In addition, T-bet controls NK cell homeostasis and, in particular, affects SP15 dependent NK cell migration (**Figure 2**). T-bet targeting could have an impact on NK cell immunotherapy in the future, and elucidating the molecular pathways of NK cell development may reveal additional targets.

### TOX

The thymocyte selection-associated HMG box factor (TOX) is a DNA-binding factor essential for T cell development (Aliahmad and Kaye, 2008). Tox has been shown to be expressed in thymocytes undergoing positive selection (Wilkinson et al., 2002) and essential for CD4 T cell development including regulatory T cells as well NKT cells but not for CD8 T cell or B cell development (Wilkinson et al., 2002; Aliahmad et al., 2004, 2011; Aliahmad and Kaye, 2008).

Interestingly, Tox^– / –^ mice have a similar phenotype to Ikaros^– / –^ and Id2^– / –^ mice, and lack mature NK cells in the periphery as well as LNs (Aliahmad et al., 2010). This study also highlights the fact that Id2 and Tox are important in the regulation of NK cell developmental stages onward to NK cell precursors and that another regulation pathway other than Tox and Id2 is required for the regulation of NK cell development. In addition, Tox has also been shown to regulate NK cell differentiation from hematopoietic stem cells *in vitro* (Yun et al., 2011).

## CONCLUDING REMARKS

Natural killer cell development in humans and mice has been widely studied for several decades. Yet, dismantling the regulation of the events that control NK cell development remains a challenge. The use of knockout mice and technology that allows the silencing of specific genes has given us a better understanding of the function and importance of TFs in NK cell development and maturation. Gaining insight into the regulation of NK cell development will unveil better strategies for NK cell systems that can be used in immunotherapy in addition to a better comprehension of the mechanisms regulating NK cells for targeted therapies.

## Conflict of Interest Statement

The authors declare that the research was conducted in the absence of any commercial or financial relationships that could be construed as a potential conflict of interest.
